# Optimisation of the techno-functional and thermal properties of heat moisture treated Bambara groundnut starch using response surface methodology

**DOI:** 10.1038/s41598-023-28451-0

**Published:** 2023-02-08

**Authors:** Vhulenda Melinda Mathobo, Oluwatoyin Oladayo Onipe, Henry Silungwe, Shonisani Eugenia Ramashia, Tonna Ashim Anyasi

**Affiliations:** 1 Department of Food Science and Technology, Faculty of Science, Engineering and Agriculture, University of Venda, Private Bag X5050, Thohoyandou, 0950 Limpopo South Africa; 2 Agro-Processing and Postharvest Technologies Division, Agricultural Research Council–Tropical and Subtropical Crops, Private Bag X11208, Nelspruit, 1200 South Africa; 3 Faculty of Applied Sciences and Biotechnology, School of Bioengineering & Food Technology, Shoolini University India, Bajhol, 173229 India; 4 Present Address: Food and Markets Department, Natural Resources Institute, University of Greenwich, Kent, ME4 4TB UK

**Keywords:** Biophysics, Biochemistry

## Abstract

This work optimised the techno-functional and thermal properties of heat moisture treated Bambara groundnut starch (BGS). A central composite rotatable design (Design-Expert software v8.0.1.0) comprising two independent factors of temperature and time was used. Extracted BGS were subjected to heat-moisture treatment (HMT) at 80–120 °C for 30–90 min at different moisture levels of 15% (HMT 15-BGS), 25% (HMT 25-BGS) and 35% (HMT 35-BGS). The optimum HMT conditions for BGS were found to be 80 °C for 30 min (HMT 15), 105.74 °C for 30 min (HMT 25), and 113.16 °C for 30 min (HMT 35). The desirability values of the obtained optimum conditions were 0.63 (HMT 15) and 1.00 (HMT 25 and 35). In HMT 35-BGS, water absorption capacity was significantly affected by the quadratic effect of temperature and time. In contrast, solubility was significantly affected by the linear effect of time and the quadratic effect of temperature. Temperature and treatment time had no significant effect (p ≥ 0.05) on the differential scanning calorimetry thermal properties of HMT 15, 25 and 35-BGS. Scanning electron micrographs of optimised BGS showed round and oval-shaped starch granules ranging from 4.2 to 4.7 mm (width) and 10 μm for length. Unmodified and optimised HMT–BGS showed characteristic FTIR bands linked with common starches. All BGS samples displayed multiple vibrations in the region below 1000 cm^−1^ due to the skeletal vibrations of the glucose pyranose ring.

## Introduction

Bambara groundnut (BG) is a self-pollinating legume preferred by farmers living in rural areas with limited resources^1^. The crop is tolerant to drought, pests and produces a reasonable yield when cultivated under poor soil conditions^2^. BG is resilient to adverse environmental conditions and can tolerate low rainfall and low fertility soils^1,3^. BG is related to cowpeas and is botanically known as *Vigna subterranea* (L.) Verdc. A member of the *Fabaceae* family. The crop is essentially grown for human consumption as it is sometimes referred to as a ‘complete food’ because it is a good supplement for cereal-based diets and a good source of plant-based protein^4,5^. The mature dried BG seeds can be boiled and eaten as a pulse; consumed whole or split and mixed with maize or plantains, milled into flour, spiced, and steamed^6^. Bambara groundnut can be processed to develop acceptable flour, paste, slurry, and a complimentary ingredient for maize porridge and shelf-stable products^7,8^. Furthermore, the legume has been utilised in the manufacture of different products, including plant-based milk^9^, low-fat yoghurt^10^, puddings^11^, protein hydrolysate as well as peptide fractions^12^.

Halimi et al*.*^5^ and Tan et al.^13^ demonstrated that BG contains 64.4% d.w. of carbohydrate, thus making it the most abundant nutrient in the seed. Most of the carbohydrate fraction in BG seed consists of complex oligosaccharides and polysaccharides, with starch comprising 33.4–53.3% of the total carbohydrates^5,13^. Starch contributes significantly to the textural properties of different food products and finds application as a thickener, colloidal stabilizer, gelling, bulking and water retention agent^14,15^. Bambara groundnut starch (BGS) contains an amylose content of between 15.7 and 35.3% depending on the seed coat colour^5,16^, with its starch granules being oval and round (A-type pattern) in shape. The starch shows a two-stage swelling pattern with a similar viscosity profile to cereal starches^17^. It also exhibits good resistance to acid at a pH range of between 4.6 and 7.0^18^. BGS displays higher swelling power, breakdown and setback but a lower gelatinisation temperature, pasting temperature, water, and oil absorption capacity^17^.

Modified starches, nonstarch hydrocolloids, emulsifiers and other food additives have been mixed with non-gluten flours to increase their baking effect^19–22^. Despite the array of starch applications obtainable, native (unmodified) starches are limited due to their intrinsic imperfect nature: insoluble in water and propensity to retrograde and endure syneresis, thus forming wabbly paste and gel^23^. Starch characteristics can be improved through the meticulous application of heat and moisture, resulting in physical changes within the starch granules^24^. Heat-moisture treatment (HMT) a safe, cheap and eco-friendly modification technique^15,25^, controls the starch-starch and starch-non-starch molecular interactions within flour, with the goal of creating modified flour for precise application in food systems^26,27^. Modification of starch using HMT thus ensures that its physicochemical characteristics are altered without triggering variations in its molecular structure^15^. The improved starches can be advantageous for nutritive purposes given the reduced digestibility arising from HMT^28,29^. Furthermore, heat treatment of all classes incapacitates anti-nutritional enzymes and enhances the flavour and general suitability of the resulting food product^15^. However, these demonstrated effects are reliant on the hydrothermal conditions as well as the botanical source or specie of the starch-containing food product^26^.

Though containing a high carbohydrate concentration, BGS requires modification techniques such as HMT, to diversify its functionality^30,31^. This is more so as unmodified BGS displays lower water and oil absorption capacity^17,29^; with their positive characteristics needed in baking and other food formulations. Several authors have established that modification of gluten-free flours and starches through hydrothermal techniques increase their physicochemical and bakery potentials^27,32,33^. Oyeyinka et al.^31^ demonstrated that modified BGS using a combination of physical and chemical techniques displayed improved functional properties. Afolabi et al.^29^ reported an increase in the gelatinization profile, swelling and solubility properties of heat-moisture treated BGS. Though these reported studies were conducted to validate the application of BGS, information on the functional and thermal properties of heat-moisture treated BGS under optimized conditions is scarce. This work therefore optimised the techno-functional and thermal properties of heat moisture treated BGS using response surface methodology.

## Materials and methods

### Source of materials

Matured dried BG seeds used for this study was obtained from Tshimbupfe in Limpopo Province of South Africa. The crop was planted in the first week of January 2017 and harvested upon maturation in the second week of April 2018. Upon harvest, the seeds were transported to the Food Science and Technology Laboratory of the University of Venda, after which the seeds were sun-dried, sorted, cleaned and used for starch extraction. The use of BG seeds in this study complies with international and national guidelines for the use of plant seeds in the study.

### Experimental design

A central composite rotatable design comprising of two independent factors of heat treatment (temperature and time) was used for the study. Dependent/response variables for this study consists of colour characteristics, functional properties, and thermal properties. Table 1 shows the upper and lower limits of the independent factors as required for the generation of experimental runs for the study. The central composite rotatable design was generated using Design-Expert software.

**Table 1:** Levels of independent variables used for central composite rotatable design.

Sample	Code	Temperature (°C)	Time (min)
Bambara starch	−1	80	30
	1	120	90

### Starch extraction and heat-moisture treatment

**Figure 1:**
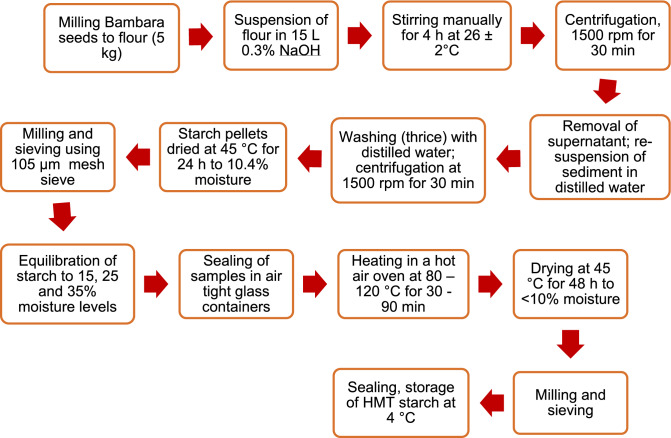
Bambara groundnut starch extraction and heat-moisture treatment of the extracted starch.

Starch was extracted from Bambara groundnut seeds^34^ using the wet milling processing technique of Hoover et al.^35^. The extracted BGS was subjected to HMT using the methods of Kittipongpatana and Kittipongpatana^36^ and Sacilik et al*.*^37^. The starch extraction method and the HMT technique used for the BGS are fully described in a flow chart in Fig. 1.

### Colour characteristics and whiteness index of Bambara groundnut starch

Colour characteristics of heat moisture treated BGS were determined with the Hunterlab Lab Scan, XE Spectrophotometer, CIELAB colour scale with the parameters L*, a*, b*, C*, H° and colour difference (∆E). Where L* designates lightness, 0–100 with 0 defining black and 100 defining white. Coordinate a* designates red (positive values) and green (negative values) while b* designates yellow (positive values) and blue (negative values)^38^. H° (hue angle) indicates the quality of lightness or darkness, while C* (Chroma) represents the quality of colour purity. The C*, H°, ΔE and whiteness index (WI) of the starch samples were obtained from L*, a*, and b* values using Eqs. (1–4).

1 $$\mathrm{Chroma }(\mathrm{C}*) =\sqrt{({a*)}^{2}+ ({b*)}^{2}}$$

2 $$\mathrm{Hue }(\mathrm{H}^\circ ) ={tan}^{-1}\left\{\frac{b*}{a*} \right\}$$

3 $$\mathrm{\Delta E }=\sqrt{{(\mathrm{\Delta L})}^{2}+{(\Delta a)}^{2}+(\Delta {b)}^{2}}$$

4 $$WI = \sqrt {\left( {100 - L*} \right)^{2} + a*^{2} + b*^{2} }$$

### Functional properties of Bambara groundnut starch

#### Water absorption capacity

Determination of water absorption capacity (WAC) was achieved by mixing 1 g (dry basis, d.b.) BGS with distilled water (10 mL) in a centrifuge tube. Subsequently, the sample was allowed to stand at room temperature (26 ± 2 °C) for 1 h, after that, centrifuged at 1500 rpm for 30 min. The volume of supernatant was measured, and WAC was calculated using Eq. (5)^39^.

5 $$\mathrm{\%WAC }=\frac{\mathrm{Volume \; of \; absorbed \; water}\times 100}{\mathrm{weight \; of \; sample}}$$

#### Oil absorption capacity (OAC)

The OAC was determined by mixing 1 g (d.b.) BGS with sunflower vegetable oil (10 mL) in a centrifuge tube. Subsequently, the sample was allowed to stand at room temperature (26 ± 2 °C) and then centrifuged at 1500 rpm for 30 min. The resultant supernatant was weighed, and OAC was calculated using Eq. 6.

6 $$\% {\text{OAC}} = \frac{{{\text{Volume}}\;{\text{of}}\;{\text{absorbed}}\;{\text{oil}} \times 100}}{{{\text{weight}}\;{\text{of}}\;{\text{sample}}}}$$

#### Swelling power and solubility

Determination of swelling power (SP) and solubility was achieved by mixing 0.6 g (d.b.) BGS with distilled water (30 mL). It was then placed in a water bath at 85 °C for 30 min, with occasional stirring and allowed to cool at room temperature (26 ± 2 °C). The mixture was centrifuged at 3000 rpm for 15 min. Subsequently, the supernatant was prudently removed, and the swollen starch sediment was measured and recorded as M_1_. The supernatant was then evaporated and dried at 105 °C overnight in a drying oven (Prolab Instrument–model OTE 80, Vancouver, Canada) until a constant weight was obtained and recorded as M_2_. Swelling power (SP) and solubility were calculated using Eqs. (7) and (8) as described by Sankhon et al.^40^.

7 $$SP = \frac{{M_{1} }}{{M_{0} }}$$

8 $${\text{Solubility }}\% \, = \frac{{M_{2} }}{{M_{0} }} \times 100$$

where M_o_ = the initial dry weight of the starch sample

### Thermal analysis

A differential scanning calorimeter (DSC, DSC 4000, Perkin-Elmer, Shelton, CT, USA) was utilised to obtain the thermal properties of BGS. The gelatinisation temperatures of onset, peak, concluding temperature, and gelatinisation enthalpy was determined as described by Arns et al.^28^. A total of 2.5 mg of heat moisture treated BGS was placed in an aluminium pan, and distilled water was added to attain a starch–water ratio of 1:3 (g/g). The pan was sealed and left to equilibrate overnight before analysis. The sample pans were then heated from 20 to 120 °C at a rate of 10 °C min^−1^. The onset, peak, concluding temperature and enthalpy change obtained using the Pyris software (Perkin-Elmer, Shelton, CT, USA) were recorded in triplicate.

### Scanning electron microscopy (SEM)

The granule structure of heat moisture treated BGS was examined using a scanning electron microscope. Heat moisture treated BGS were attached to SEM stubs with a double-sided carbon tape and gold-coated using an Edwards S150A sputter-coater to enhance conductivity. The modified starch was then visualised with a Zeiss Merlin Field Emission Scanning Electron Microscope (FESEM, Carl Zeiss Microscopy, Germany). SEM images were generated with the aid of Zeiss In Lens SE (Secondary Electron) and SE2 detectors and Zeiss Smart SEM software at 3 kV accelerating voltage and 100 pA beam current^41^ with a working distance of 4.2 to 4.8 mm and magnification of 100×.

### Fourier transform infrared spectroscopy

Fourier transform infrared spectroscopy (FTIR) spectra of unmodified and heat-moisture treated BGS were attained using a Bruker Alpha FTIR spectrophotometer (120HR, Bruker Alpha, Germany). The background spectra of the instrument were recorded before 0.5 g of starch samples was mounted onto the instrument. The spectra were noted with characteristic peaks in wavenumbers from 450 to 4000 cm^−1^ at 16 runs per scan^41^.

### Statistical analysis

All experiments were conducted in triplicate. Analysis of variance (ANOVA), regression models, optimisation and contour plots response surface graphs were carried out using Design-Expert software version 8.0.1.0 (Stat-Ease, Minneapolis, MN 55413, USA).

## Results and discussion

### Colour characteristics and whiteness index of heat moisture treated Bambara groundnut starch

The colour characteristics of heat moisture treated BGS under different HMT conditions of moisture (15, 25 and 35%), temperature (71.72–128.28 °C) and time (17.57–102.43 min) are shown in Table 2. Colour is a significant attribute commonly evaluated by consumers when they buy foods and influences consumption patterns. The L* value is defined as the psychometric index of lightness, and a higher whiteness value of starch is ideal for consumer acceptability^42^. The whiteness index (WI) suggests the whiteness of a food product and shows the degree of discolouration during the treatment process^43^. The colour characteristics of HMT 15-BGS ranged from 79.3–82.3 (L*), 1.2–2.5 (a*), 3.1–4.7 (b*), 3.3–5.2 (C*), 60.2–69 (H°), 79–81.9 (WI) and 18–21.9 (∆E). The colour characteristics of HMT 25-BGS ranged from 78.4–82 (L*), 1.6 -2.3 (a*), 3.3–4.5 (b*), 3.6–5.1 (C*), 59.1–66.6 (H°), 77.8–81.6 (WI) and 18.5–22.3 (∆E). HMT 35-BGS colour characteristics ranged from 74.2–80.8 (L*), 1.6–4.4 (a*), 4.2–5.8 (b*), 4.5–7.3 (C*), 53.2–71.1 (H°), 73.2–80.3 (WI) and 19.7–27.3 (∆E). The minor inconsistencies in the colour characteristics of the starch samples were expected as dissimilar HMT conditions (combination of temperature and time) were applied. In this study, colour characteristics in terms of L* and WI were considered as they are more applicable to starch.

**Table 2:** Levels of process variables and values for colour characteristics of heat-moisture treated Bambara groundnut under different treatment conditions.

Independent variables		Response variables						
Temperature (°C)	Time (min)	L*	a*	b*	C*	H°	WI	∆E
HMT 15								
100	17.57	81.9	1.5	3.4	3.7	66.6	81.5	19.0
100	60	80.7	2.5	4.6	5.2	61.6	80.0	20.1
100	60	82.2	1.8	3.3	3.7	61.0	81.8	18.6
100	102.43	79.3	1.2	3.1	3.3	68.2	79.0	21.9
120	30	81.8	1.8	3.6	4.0	63.9	81.4	19.5
120	90	80.6	2.2	3.9	4.5	60.2	80.1	20.1
80	30	82.3	1.3	3.2	3.5	68.0	81.9	18.0
80	90	82.1	1.4	3.5	3.8	69.0	81.7	18.4
71.72	60	81.8	1.7	3.1	3.6	62.0	81.5	18.4
128.28	60	81.8	1.9	4.7	5.0	68.1	81.1	19.0
HMT 25								
100	17.57	81.5	1.8	3.4	3.8	61.7	81.1	18.9
100	60	81.1	1.7	3.6	4.0	64.4	80.7	19.4
100	60	82.0	1.8	3.6	4.0	64.3	81.6	19.1
100	102.43	80.2	2.3	4.2	4.7	60.2	79.6	19.9
120	30	81.8	1.6	3.3	3.6	64.6	81.4	18.6
120	90	78.4	2.3	4.5	5.1	63.5	77.8	22.3
80	30	81.3	1.9	3.8	4.2	63.3	80.8	19.7
80	90	81.2	1.8	3.4	3.8	61.3	80.8	18.5
71.72	60	81.2	1.7	4.0	4.4	66.6	80.7	18.9
128.28	60	80.5	2.3	3.9	4.6	59.1	79.9	20.1
HMT 35								
100	17.57	80.8	1.7	4.2	4.5	67.9	80.3	19.7
100	60	75.7	3.6	5.8	6.9	58.1	74.8	23.7
100	60	77.9	2.4	4.3	4.9	61.0	77.4	23.2
100	102.43	77.8	3.2	5.3	6.2	59.1	76.9	22.7
120	30	79.7	2.4	5.0	5.5	64.6	78.9	20.3
120	90	74.2	4.4	5.8	7.3	53.2	73.2	27.3
80	30	80.1	2.4	4.3	4.9	60.9	79.5	21.0
80	90	78.9	2.0	4.7	5.1	67.0	78.3	21.0
71.72	60	80.6	1.6	4.8	5.1	71.1	79.9	20.0
128.28	60	75.6	2.1	4.8	5.2	66.6	75.0	23.7

L*: lightness/darkness; a*: redness/greenness; b*: yellowness/blueness; C*: chroma; H°: hue angle; WI: whiteness index; ∆E: total colour difference. Total colour difference (ΔE) is a noticeable difference, where the observer does not notice the difference^38^.

**Figure 2:**
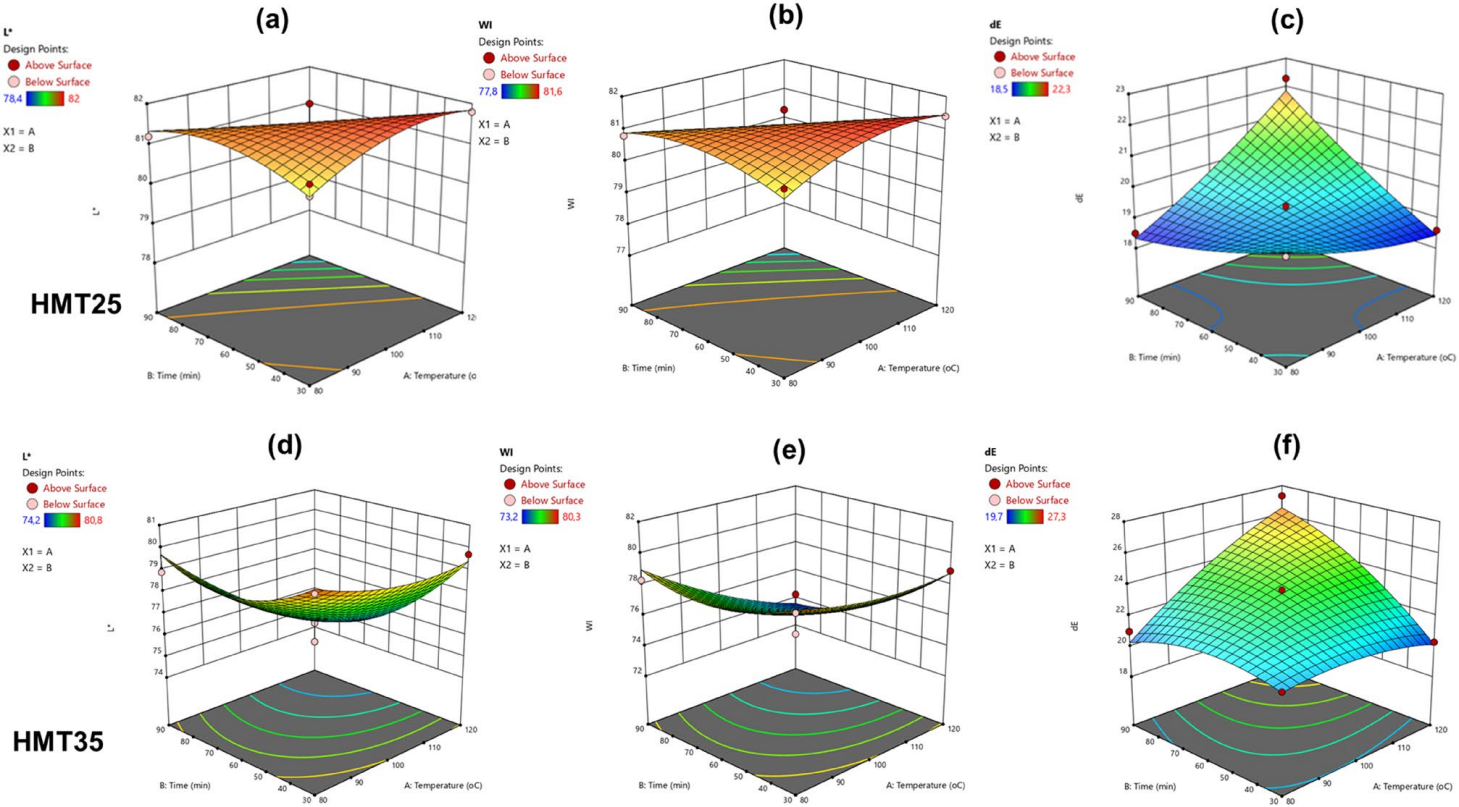
Response surface plots illustrating the effects of treatment temperature and time on Lightness (L*), whiteness index (WI) and colour change (∆E) of HMT-25 (**a**–**c**) and L*, WI and ∆E of HMT-35 Bambara groundnut starch.

The highest L* and WI values for heat moisture treated BGS were observed at 80 °C for 30 min (HMT 15-BGS); 100 °C for 60 min (HMT 25-BGS), and 100 °C for 17.57 min (HMT 35-BGS) while the lowest was observed at HMT 100 °C for 102.43 min (15% MC); 120 °C for 90 min (25% MC) and 120 °C for 90 min (35% MC). The low a* and b* values obtained for all heat moisture treated BGS suggest that samples were less red and yellow, justifying the moderately higher WI and L* values of the starch samples obtained in this study. Analysis of variance (ANOVA) of the effect of model parameters on colour characteristics of HMT-BG starch showed that effects of temperature and treatment time had no significant (p ≥ 0.05) effect on the colour characteristics (L*, a*, b*, C*, H°, WI, ∆E) of HMT 15-BGS, but significant model terms (p ≤ 0.05) for all colour characteristics of HMT 25-BGS and L* and WI of HMT 35 starch samples. The linear terms of time had a significant effect (p < 0.05) on L*, a*, b*, C and WI of the HBG-25 BGS samples. The regression models for colour characteristics of HMT-BGS (Table 3) are characterised by a non-significant lack of fit (p ≥ 0.05). Non-significant lack of fit is good as it guarantees the models fit the experimental data, and there is a significant effect on parameters on output response. Response surface plots illustrating the effects of HMT treatment temperature and time on L*, WI and total colour difference (∆E) of HMT 25-BGS and HMT 35-BGS are presented in Fig. 2. In HMT 25-BGS, L* and WI increased with an increase in heating temperature (Fig. 2a,b), while a rise in ∆E resulted from increasing temperature and decreasing time (Fig. 2c). In HMT 35-BGS, the L* and WI of the samples increased as treatment time and temperature decreased, ∆E of the starch samples increased with an increase in HMT treatment temperature and time (Fig. 2d–f).

**Table 3:** Regression models relating colour characteristics and model parameters of heat-moisture treated Bambara groundnut starch.

	L*	a*	b*	C*	H°	WI	∆E
HMT15							
Intercept	+ 81.45	+ 2.15	+ 3.95	+ 4.45	+ 61.30	+ 80.90	+ 19.35
A	− 0.2500	+ 0.1625	+ 0.1000	+ 0.1500	− 1.61	− 0.2625	+ 0.5061
B	− 0.6346	+ 0.0095	+ 0.0220	+ 0.0293	− 0.0547	− 0.6294	+ 0.6377
AB	− 0.2500	+ 0.0750	+ 0.0000	+ 0.0500	− 1.17	− 0.2750	+ 0.0500
A^2^	+ 0.3000	− 0.1875	− 0.3313	− 0.3250	+ 0.4938	+ 0.4000	− 0.4687
B^2^	− 0.3000	− 0.3625	− 0.2563	− 0.3750	+ 3.19	− 0.2250	+ 0.4062
Lack of fit	0.7522^	0.7501^	0.8920^	0.8788^	0.1085^	0.8418^	0.6663^
R^2^	0.6612	0.5804	0.3300	0.3478	0.7576	0.6519	0.6949
HMT25							
Intercept	+ 81.55	+ 1.92	+ 3.60	+ 4.00	+ 64.35	+ 81.15	+ 19.25
A	− 0.4112	+ 0.1311	+ 0.0573	+ 0.1229	− 0.8883	− 0.4414	+ 0.5496
B	− 0.6673	+ 0.1634	+ 0.2414	+ 0.2966	− 0.6527	− 0.7152	+ 0.4893
AB	− 0.8250	+ 0.2000	+ 0.4000	+ 0.4750	+ 0.2250	− 0.9000	+ 1.23
A^2^	− 0.3937	−	+ 0.1437	+ 0.2000	− 0.4313	− 0.4562	+ 0.2062
B^2^	− 0.3937	−	+ 0.0688	+ 0.0750	− 1.38	− 0.4312	+ 0.1562
Lack of fit	0.7437^	0.2911^			0.0164	0.7811^	0.3659^
R^2^	0.8938	0.7559	0.9152	0.9477	0.3994	0.9177	0.9522
HMT35							
Intercept	+ 76.80	+ 3.00	+ 5.05	+ 5.90	+ 59.55	+ 76.10	+ 23.45
A	− 1.52	+ 0.3884	+ 0.2250	+ 0.3677	− 2.06	− 1.58	+ 1.35
B	− 1.37	+ 0.4652	+ 0.3445	+ 0.5505	− 2.22	− 1.46	+ 1.41
AB	− 1.07	+ 0.6000	+ 0.1000	+ 0.4000	− 4.37	− 1.12	+ 1.75
A^2^	+ 0.5313	− 0.4125	− 0.0813	− 0.2625	+ 3.46	+ 0.5375	− 0.5813
B^2^	+ 1.13	− 0.1125	− 0.1063	− 0.1625	+ 0.7875	+ 1.11	− 0.9062
Lack of fit	0.8700^	0.7219^	0.9227^	0.8783^	0.3402^	0.9293^	0.2757^
R^2^	0.9145	0.7257	0.4748	0.5749	0.7830	0.9101	0.9468

*A:* linear effect of treatment temperature*; B:* linear effect of treatment time*; AB:* interaction of temperature and treatment time*; A*^2^*:* quadratic effect of temperature*; B*^2^*:* quadratic effect of treatment time; L*: lightness/darkness; a*: redness/greenness; b*: yellowness/blueness; C*: chroma; H°: hue angle; WI: whiteness index; ∆E: total colour difference.

^^^Not significant at p ≥ 0.05.

Barua and Srivastav^42^ reported L*, a* and b* values ranging between 83.66–87.68, 1.73–2.16 and 14.24–16.74, respectively, on mung bean starch heat moisture treated at 30% moisture, for 16 h at 80–120 °C. The L* (63.71), a* (4.19), b* (10.28) values, H° (81.20) and total colour difference (7.51) have also been reported for HMT potato starch with 20–25% at 120 °C^44^. Liestianty et al*.*^45^ showed colour characteristics of heat moisture treated sago starch at 28% moisture and 110 °C for 2–6 h. The authors observed that L*, a*, and b* values ranged from 80.58–82.17, 0.75–1.25 and 6.86–7.41, respectively. Nadir et al*.*^44^ reported a decrease in L* and b* values with an increase in a* value, but a notable reduction in Hue angle and colour difference after HMT. It can be observed that different HMT conditions have varying effects on colour properties. It has been reported that changes in colour characteristics of starches during physical methods like HMT could be due to the separation and purification of heterogeneous materials, including sugars, proteins, salt, and other elements^10,44^. Variations in colour are often attributed to the maillard reactions occurring between the amino acid groups in protein as well as the reducing sugars in starch^15,46^. Barua and Srivastav^42^ stated that the observed changes in colour may be due to caramelization reaction which produces simple sugars as a result of the breaking down of the starch molecules.

### Functional properties of heat moisture treated Bambara groundnut starch

Application of HMT to BGS is dependent on its functionality which can be deduced from characteristics such as WAC, OAC, SP and solubility of the starch. The functional properties of BGS treated under different HMT conditions are shown in Table 4.

**Table 4:** Levels of process variables and values of functional properties for heat-moisture treated Bambara groundnut under different treatment conditions.

Independent variables		Response variables			
Temperature (°C)	Time (min)	WAC (%)	OAC (%)	SP (g/g)	Solubility (%)
HMT 15					
100	17.57	200	180	12.2	20
100	60	190	240	12.2	10
100	60	170	180	11.2	11.7
100	102.43	200	240	8.7	11.7
120	30	190	200	11.7	21.7
120	90	200	240	11.6	18.3
80	30	180	200	13.7	16.7
80	90	200	240	7	15
71.72	60	180	280	13.3	10
128.28	60	190	200	10.8	10
HMT 25					
100	17.57	210	320	8	13.3
100	60	190	300	8.2	18.3
100	60	180	300	8.5	15
100	102.43	170	320	7.9	15
120	30	170	220	10.7	18.3
120	90	190	200	8	8.3
80	30	210	300	12.2	5
80	90	180	200	4.5	5
71.72	60	180	200	13.2	15
128.28	60	170	220	9.7	10
HMT 35					
100	17.57	170	200	9.2	8.3
100	60	200	300	6.25	11.7
100	60	260	200	7.0	6.7
100	102.43	180	260	6.3	8.3
120	30	200	280	7.8	11.7
120	90	290	100	7	5
80	30	200	280	7.5	13.3
80	90	210	240	7.9	15
71.72	60	100	300	9.9	13.3
128.28	60	290	240	8	8.3

WAC: water absorption capacity; OAC: oil absorption capacity; SP: swelling power.

The WAC and OAC of starch is the ability of starch to take up water or oil^47^. WAC indicates the water-binding and emulsifying potential of that specific starch^39,47^. WAC is a function of numerous strictures, including hydrogen bonds, degree of accessibility of water binding sites among the starches, conformational characteristics, stearic factors, and hydrophilic–hydrophobic balance^48,49^. Hoover and Sosulski^50^ showed that water-binding could be affected by the physical and chemical environment of starch suspensions, including pH, ionic strength, vapour pressure, temperature, and the presence or absence of a surfactant. Temperature greatly affects water absorption of starch because when the temperature rises, the molecules obtain sufficient thermal energy. The thermal energy weakens the intermolecular hydrogen bonds and increases interaction and affinity towards the water; as a result, the starch granules integrate with water^51^.

Swelling power is the hydration ability of starch at elevated temperatures. When starch is heated in water, the granules absorb water and swell; the attained thermal energy then weakens the inner bonds and binding forces of the starch granule. The swelling and solubility of the starches are directly influenced by the proportion of the crystalline and amorphous components of the starch granules^48^. High swelling and solubility index are ascribed to the moveable granule structure, low molecular weight of amylose, amylopectin ratio and molecular features such as chain length, branching, micellar granular arrangement and the presence of lipids that form complexes^52^. Adebowale et al*.*^53^, in a study on the functional, physicochemical and retrogradation features of sword beans (*Canavalia gladiata*) acetylated and oxidized starch, observed that starch granules with a wide and strongly bonded micellar structure show a comparatively great resistance towards swelling, which may eventually result to a lesser solubility index.

The swelling power of starch from different legume sources is positively affected by the temperature and pH of the aqueous starch slurries. This is because thermal energy relaxes the strong intra-granular interactions, which disrupts the molecules of starch granules^35,49^. Adebowale and Lawal^54^ reported that legume starches displayed improved functional properties than cereal and potato-based starches. It can be speculated that legume starches may attain a comparable degree of swelling and solubility as cereal starches at higher temperatures. The swelling power and solubility index indicates the extent of interactions between starch chains in the amorphous and crystalline region.

The highest values of OAC, SP and solubility of BGS with 15% MC were obtained at HMT 71.72 °C for 60 min, 80 °C for 30 min and 120 °C for 30 min respectively, whereas the highest values for WAC were obtained at HMT 100 °C for 17.57 min; 100 °C for 102.43 min; 80 °C for 90 min; and 120 °C for 90 min. Furthermore, the highest values of WAC, OAC, SP and solubility of BGS with 25% MC were obtained at HMT 100 °C 17.57 min and 80 °C for 30 min; 100 °C for 17.57 min and 100 °C for 102.43 min; 71.72 °C for 60 min and 100 °C for 60 min; as well as 120 °C for 30 min respectively. Consequently, the highest values of WAC, AOC, swelling power and solubility of BG starch with 35% MC were obtained at HMT 120 °C for 90 min and 128.28 °C for 60 min; 100 °C for 60 min and 71.72 °C for 60 min; 71.72 °C for 60 min and 80 °C for 30 min; as well as HMT 71.72 °C for 60 min respectively. Treatment time had a significant linear effect (p ≤ 0.05) on swelling power for HMT 15-BGS. In HMT 35-BGS, WAC was significantly affected by the quadratic effect of temperature and time, while solubility was significantly affected by the linear effect of time and the quadratic effect of temperature. However, temperature and treatment time had no significant (p ≥ 0.05) impact on the functional properties (OAC, SP and solubility) of the HMT 25- BGS.

A comparison of the functional properties of heat moisture treated starches from different sources, and the present study is shown in Table 5. The Table reflects disparities in findings obtained from the literature and the present study. These disparities could be attributed to the rearrangement of molecular chains during modification and strengthening inner forces, which prohibits water absorption within starch matrices. HMT has been reported to cause some alterations in the crystalline and amorphous regions of starch granules. Amylose content and starch chain length is a factor that significantly affects the functional properties of the final product^58,61–64^. The hydrophilic group of modified starches is more capable of binding with water and, therefore, facilitates water absorption^39^. The regression models for predicting the functional properties of heat moisture treated BGS is shown in Table 6. The regression models for these functional properties of HMT-BGS are characterised by a non-significant lack of fit (p ≥ 0.05). Non-significant lack of fit is good as this guarantees a good fit for the experimental data models.

**Table 5:** Comparison of some functional properties of various heat-moisture treated starches in previous and present studies.

Source of starch	Moisture level (%)	WAC (%)	OAC (%)	SP (g/g)	Solubility (%)	References
Present study						
BG	15	170–200	180–280	7–13.7	10–21.7	
BG	25	170–210	200–320	4.5–13.2	5–18.3	
BG	35	100–290	100–300	6.25–9.9	5–13.3	
Previous studies						
Buckwheat	25	152.74	167.42	23.27	11.96	^55^
Locust bean	20	–	–	5.57	39.5	^40^
Pinhao	15–25	–	–	9.54–13.96	5.19–10.08	^56^
Arrowroot	28			19.98–26.64	11.8–20.77	^57^
White sorghum	18–27	220–380	140–180	4.04–8.11	0.54–5.0	^58^
Potato	20–25	0.78	0.57	44.92	12.67	^44^
Rice	22	–	–	11.1–14.07	6.36–6.99	^59^
Cassava	22	–	–	13.57–17.80	4.40–5.47	^59^
Jack fruit seed	10–35	–	–	1.2–7.0	1.5–9.6	^36^
Sweet potato	30	1.29	–	1.67	1.97	^39^
Mung bean	30	–	–	9.4–10.94	5.22–6.58	^42^
Finger millet	20–30	3.05–3.25	2.04–2.50	180–300	3–14	^60^

WAC: water absorption capacity; OAC: oil absorption capacity; SP: swelling power; BG: Bambara groundnut.

**Table 6:** Regression models relating functional properties and model parameters of heat-moisture treated Bambara groundnut starch.

	WAC	OAC	SP	Solubility
HMT15				
Intercept	+ 180.00	+ 210.00	+ 11.70	+ 10.85
A	+ 3.02	− 14.14	− 0.1169	+ 1.04
B	+ 3.75	+ 20.61	− 1.47	− 2.10
AB	− 2.50	+ 0.0000	+ 1.65	− 0.4250
A^2^	+ 2.50	+ 13.75	+ 0.1125	+ 0.8250
B^2^	+ 10.00	− 1.25	− 0.6875	+ 3.75
Lack of fit	0.8938^	0.8316^	0.3697^	0.1883^
R^2^	0.6854	0.6403	0.8448	0.6318
HMT25				
Intercept	+ 185.00	+ 300.00	+ 8.35	+ 16.65
A	− 5.52	− 6.46	− 0.3687	+ 1.19
B	− 8.32	− 15.00	− 1.32	− 0.9495
AB	+ 12.50	+ 20.00	+ 1.25	− 2.50
A^2^	− 3.75	− 53.75	+ 1.34	− 3.12
B^2^	+ 3.75	+ 1.25	− 0.4125	− 2.29
Lack of fit	0.4393^		0.0594	0.2592^
R^2^	0.8037	0.7798	0.6275	0.4182
HMT35				
Intercept	+ 230.00	+ 250.00	+ 6.63	+ 9.20
A	+ 43.59	− 28.11	− 0.4109	− 2.33
B	+ 14.27	− 16.89	− 0.5627	− 0.6250
AB	+ 20.00	− 35.00	− 0.3000	− 2.10
A^2^	− 7.50	+ 3.75	+ 0.9625	+ 1.23
B^2^	− 17.50	− 16.25	+ 0.3625	− 0.0250
Lack of fit	0.5146^	0.6347^	0.3413^	0.8317^
R^2^	0.6565	0.4569	0.6898	0.7526

*A:* linear effect of treatment temperature*; B:* linear effect of treatment time*; AB:* interaction of temperature and treatment time*; A*^2^: quadratic effect of treatment temperature*; B*^2^: quadratic effect of treatment time; WAC: water absorption capacity; OAC: oil absorption capacity; SP: swelling power.

^^^Not significant at p ≥ 0.05.

Chung et al.^65^, Gunaratne and Hoover^63^, Senanayake et al.^66^,Olayinka et al.^58^, Zeng et al.^64^, Sharma et al*.*^47^ and Adebowale et al*.*^60^ all reported a decrease in swelling power and solubility for corn, cassava, sweet potato, peas, sorghum, waxy rice, pearl and finger millet starches respectively, due to the application of HMT. The decrease in solubility suggests a strengthening of bonds and an increase of interactions among amylopectin-amylopectin molecules, thereby slowing them down from leaching out of the starch granules. Consequently, the reduction in swelling power by HMT is caused by the internal reorganisation of starch granules, resulting in an interaction between starch functional groups, making it form a more ordered double-helical amylopectin side-chain cluster^67^. The decrease in swelling capacity can be attributed to the structural re-association of starch chains caused by the HMT and thus restriction for hydration, while starch solubility results from the leaching of amylose, which separates from starch granules and thus spreads out of starch granules during swelling^68^.

Sarkar^55^ reported increasing WAC and OAC in HMT buckwheat starch. Water absorption capacity may increase in the starch when amylose and amylopectin are loosely associated^55^. Nadir et al*.*^44^ also reported high WAC but a decrease in OAC by HMT in potato starch. This variation by HMT is due to changes in the hydrophobic and hydrophilic tendencies of the starch, which affects the oil and water absorption capacity of the starch^61^. The hydrophobic sites of starch promote oil absorption, while the hydrophilic site of the starch promotes water absorption^61,69^. Therefore, the ability of starch to absorb oil and water is a good indication of the emulsifying potentials of the starch^60^.

### Differential scanning calorimetry

Starch gelatinisation is an endothermic alteration significant for separating amylopectin double helices from the ordered structure to a disordered one^70^. This phenomenon is significant in processing techniques, forming unique textural and structural characteristics in food products. Gelatinisation temperature also characterizes the starch type and is dependent on the glass transition of the amorphous fraction of the starch^68^. Cheng et al.^70^ postulated that the onset temperature (*T*_*o*_) indicates the melting temperature of the weakest crystalline in starch granules, while the conclusion temperature (*T*_*c*_) is the melting temperature of high perfection crystalline. Singh et al*.*^14^ stated that the gelatinization enthalpy (ΔH) primarily represents the loss of double-helical components within starch granules. The thermal properties (gelatinization) of BG starch under different HMT conditions are presented in Table 7.

**Table 7:** Levels of process variables and values of gelatinization for heat-moisture treated Bambara groundnut under different treatment conditions.

Independent variables		Response variables			
Temperature (°C)	Time (min)	*T*_*o*_ (°C)	*T*_*p*_ (°C)	*T*_*c*_ (°C)	∆H (J/g)
HMT 15					
100	17.57	75.86	81.52	87	5.36
100	60	70.09	77.81	87.54	5.19
100	60	94.63	97.03	94.63	2.95
100	102.43	83.39	86.79	90.42	1.26
120	30	93.20	96.11	98.59	2.8
120	90	78.26	84.19	90.54	1.17
80	30	89.18	93.61	98.92	4.24
80	90	78.02	85.83	94.75	7.93
71.72	60	92.91	97.85	102.76	9.21
128.28	60	78.13	87.35	97.74	8.27
HMT 25					
100	17.57	59.12	72.21	86.86	7.73
100	60	76.40	81.26	87.1	3.57
100	60	78.58	86.58	96.97	17.90
100	102.43	71.80	81.24	89.99	3.79
120	30	62.62	75.87	92.26	1.20
120	90	76.85	90.25	104.71	18.14
80	30	77.76	90.56	102.34	11.86
80	90	87.77	96.14	100.28	12.26
71.72	60	82.14	92.63	103.23	3.48
128.28	60	92.64	99.47	102.92	12.26
HMT 35					
100	17.57	80.45	88.63	97.52	9.37
100	60	66.62	80.52	92.43	9.21
100	60	70.39	81.44	92.89	11.35
100	102.43	68.28	78.55	91.32	1.24
120	30	66.45	78.36	90.50	11.11
120	90	70.53	83.43	96.56	6.17
80	30	78.37	90.33	102.21	6.03
80	90	74.95	89.67	105.42	8.26
71.72	60	85.53	97.18	108.88	6.08
128.28	60	85.23	93.83	103.43	1.66

*T*_*o*_ (°C): onset temperature; *T*_*p*_ (°C): peak temperature; *T*_*c*_ (°C): conclusion temperature; ∆H (J/g): gelatinization enthalpy.

The highest values of *T*_*o*_ for BGS with 15% moisture content (MC) were obtained at HMT; 100 °C for 60 min while the highest values of *T*_*p*_, *T*_*c*_ and ∆H were obtained at HMT 71.72 °C for 60 min. Similarly, the highest values of *T*_*o*_ and *T*_*p*_ of BGS with 25% MC were obtained at HMT 128.28 °C for 60 min, and the highest values of *T*_*c*_ and ∆H were obtained at HMT 120 °C for 90 min. Consequently, the highest values of *T*_*o*_, *T*_*p*_ and *T*_*c*_ were obtained at HMT 71.72 °C for 60 min, while the highest ∆H values of BGS with 35% MC were obtained at HMT 100 °C for 60 min (Table 7). The effect of HMT on thermal properties is comparable to starches of other crops such as maize, cassava, millet, locust bean, lentil, yam bean and pea (Table 8). Gelatinisation temperature of starch obtained from BG has been reported as 71.69 °C at the onset temperature (*T*_*o*_), (75.33 °C) at the peak temperature (*T*_*p*_), (79.17 °C) at the gelatinization conclusion temperature (*T*_*c*_) and 11.73 (J/g) for enthalpy of gelatinization (ΔH)^72^. The higher gelatinization temperature for BGS may result from the more rigid granular starch structure^14^. This is associated with structural variations within the starch granules involving amylose-amylose and amylose–lipid interactions. These interactions reduce the mobility of the amorphous region, which result in higher temperature required for the swelling and disruption of the crystalline regions^71^.

**Table 8:** Comparison of gelatinization temperatures of the present study with previous studies.

Source of starch	Moisture level	*T*_*o*_ (°C)	*T*_*p*_ (°C)	*T*_*c*_ (°C)	∆H (J/g)	Reference
Present study						
BG	15	70.09–94.63	77.81–97.85	87–102.76	1.17–9.21	
BG	25	59.12–92.64	72.21–99.47	86.86–104.71	1.20–18.14	
BG	35	66.45–85.53	78.36–97.18	91.32–108.88	1.24–11.35	
Previous studies						
Rice	22	63.51 -70.62	73.07–74.45	73.77–76.10	1.68–6.10	^59^
Waxy maize	25	75.02–82.02	81.50–89.22	90.76–95.44	2.73–3.11	^70^
Non-waxy maize	25	75.52–83.78	79.85–89.72	86.57–96.77	2.77–2.83	^70^
African yam bean	18	73.57	76.49	79.23	0.543	^61^
African yam bean	27	75.30	81.02	86.50	0.112	^61^
Locust bean	20	63.7	82.5	103.6	8.94	^40^
Finger millet	20–30	60.21–62	65.37–66.39	63.52–71.72	0.64–1.11	^60^
Maize	30	67.8	82.2	88.8	6.1	^62^
Pea	30	64.3	88.6	97.5	7.1	^62^
Lentil	30	65.9	86.4	96.3	8.0	^62^
Jack fruit seed	35	88.27–93.26	91.14–95.45	93.40–98.05	1.63–5.04	^36^
Potato	30	61.2	75.6	86.5	11.5	^63^
Wheat	25	63.36–64.13	68.52–69.52	76.71–78.99	8.14–9.11	^71^
Pearl millet	20–30	64.5–76.2	64.6–80.8	75.5–88.7	9.6–10.8	^47^

*T*_*o*_: onset temperature; *T*_*p*_: peak temperature; *T*_*c*_: conclusion temperature; ∆H: Gelatinization enthalpy; BG: Bambara groundnut.

The observed variations in the starch gelatinization temperatures could be attributed to the effects on the molecular structure of amylopectin, starch composition, and the granular arrangement of the starch particles^63^. Furthermore, these differences in gelatinization temperatures could be due to the amylose content, size, and the re-alignment of starch helices within the starch^38^. In HMT 15-BGS, the *T*_*o*_, *T*_*p*_ and *T*_*c*_ of the samples decreased as treatment time and temperature increased (Supplementary Fig. 1). In contrast, the gelatinization enthalpy of the starch samples increased with an increase in HMT treatment temperature and time. In HMT 25-BGS, the *T*_*p*_ and gelatinization enthalpy of the starch samples increased with an increase in HMT treatment temperature and time (Supplementary Fig. 2). However, a gradual rise in *T*_*o*_ and *T*_*c*_ increased as HMT time and temperature increased. In HMT 35-BGS, the *T*_*o*_, *T*_*p*_, *T*_*c*_ and gelatinization enthalpy (∆H) of the starch samples decreased with an increase in HMT treatment temperature and time (Supplementary Fig. 3). Generally, heat moisture treated starch exhibits an increased ∆H. However, if the incubation temperature is above starch gelatinization temperature during treatment, partial gelatinization of starch and decrease in ∆H can result^73–75^. The increase in *T*_*o*_, *T*_*p*_, and *T*_*c*_ by HMT is due to amylose-amylose, amylose-amylopectin and amylose–lipid interactions. These interactions restrain the mobility of starch chains in the amorphous regions. Consequently, the amorphous regions would require a higher temperature to swell, contributing to the disruption of the crystalline regions^60,64^.

The ANOVA of the effect of model parameters on thermal properties of HMT 15-BG, HMT 25-BG and HMT 35-BGS shows that HMT treatment temperature and time had no significant (p ≥ 0.05) effect on the thermal properties of the heat moisture treated BGS. The regression models for predicting thermal properties of heat moisture treated BGS (HMT 15, 25 and 35) are shown in Table 9. The regression models for thermal properties of HMT-BGS are characterized by a non-significant lack of fit (p ≥ 0.05). The non-significant lack of fit indicates a fitness of the models for the experimental data.

**Table 9:** Regression models relating gelatinization parameters and model parameters of heat-moisture treated Bambara groundnut starch.

	*T*_*o*_ (°C)	*T*_*p*_ (°C)	*T*_*c*_ (°C)	∆H (J/g)
HMT15				
Intercept	+ 82.36	+ 87.42	+ 91.09	+ 4.07
A	− 2.08	− 1.75	− 1.45	− 1.19
B	− 1.93	− 1.53	− 0.9229	− 0.4673
AB	− 0.9450	− 1.03	− 0.9700	− 1.33
A^2^	+ 2.10	+ 2.98	+ 4.89	+ 1.84
B^2^	− 0.8444	− 1.24	− 0.8825	− 0.8775
Lack of fit	0.8427*	0.8712*	0.7355*	0.4040*
R^2^	0.1588	0.2834	0.7405	0.6801
HMT25				
Intercept	+ 77.49	+ 83.92	+ 92.04	+ 10.74
A	− 1.40	− 1.36	− 0.7611	+ 0.9546
B	+ 5.27	+ 4.09	+ 1.85	+ 1.47
AB	+ 1.06	+ 2.20	+ 3.63	+ 4.13
A^2^	+ 4.91	+ 6.52	+ 6.56	− 0.4200
B^2^	− 6.06	− 3.14	− 0.7681	− 1.48
Lack of fit	0.1331*	0.3957*	0.8401*	0.7714*
R^2^	0.7701	0.7891	0.7951	0.3024
HMT35				
Intercept	+ 68.50	+ 80.98	+ 92.66	+ 10.28
A	− 2.10	− 2.87	− 3.53	− 0.4076
B	− 2.07	− 1.23	+ 0.0627	− 1.78
AB	+ 1.88	+ 1.43	+ 0.7125	− 1.79
A^2^	+ 6.61	+ 6.24	+ 6.34	− 2.38
B^2^	+ 1.11	+ 0.2800	+ 0.4763	− 1.66
Lack of fit	0.2478*	0.0827*	0.0507*	0.2864*
R^2^	0.6184	0.7470	0.8249	0.6032

*A:* linear effect of treatment temperature*; B:* linear effect of treatment time*; AB*: interaction of temperature and treatment time*; A*^2^: quadratic effect of treatment temperature*; B*^2^: quadratic effect of treatment time; *T*_*o*_: onset temperature; *T*_*p*_: peak temperature; *T*_*c*_: conclusion temperature; ∆H: gelatinization enthalpy.

*Not significant at p ≥ 0.05.

Hoover^75^ postulated that the extent to which amylose-amylose, amylose-amylopectin, and amylose–lipid interactions associate during HMT is influenced by the starch source amylose chain length and the moisture content during HMT. An increase in gelatinization parameters on HMT has been linked to the interaction between amylose–amylose, amylose–amylopectin, amylopectin–amylopectin chains, and the formation of other complexes between starch amylose and lipids^76^. Moreover, an increase in gelatinization parameters could suggest that crystallites disrupted by HMT may have combined to form larger ones. However, Zavereze et al*.*^68^ indicated that the strength of intermolecular bonds in starch promoted by HMT requires a higher temperature to gelatinize the starch granules. Zeng et al.^64^ and Sharma et al.^47^ reported an increase in *T*_*o*_, *T*_*p*_, and *T*_*c*_ with a decrease in ∆H during HMT of waxy rice and pearl millet starch. A reduction in ∆H after HMT has also been reported for pea, navy bean, regular maize starch, lentils, and waxy potato starches^62,65,77^. Similarly, reduced ∆H promoted by HMT were reported in jack bean, corn, potato, and cassava starches^47,63,78^. The reduction in gelatinization enthalpy by HMT results from the disruption of the crystallites, which are unstable, hence leading to a lower degree of crystallinity and, therefore, requiring less energy for disruption^63^. Chung et al.^62^ stated that reduction by the HMT demonstrates that the high temperature during HMT may increase the mobility of double helices forming the crystalline structure, thereby disrupting the hydrogen bonds between the helices. Hormdok and Noomhorm^79^ showed that the reduction in gelatinization after HMT may be due to the partial gelatinization of amylose and amylopectin molecules, which becomes highly unstable during heating. Cheng et al*.*^70^ reported that HMT at 25% moisture had a significant effect on gelatinization parameters of maize starch. However, the findings of this present study negate the observation of Cheng et al*.*^70^. This may be due to the differences in HMT conditions used in Cheng et al.^70^ (120 °C, 3–9 h) and starch origin used in the present study.

### Correlation analysis

Correlation analysis was conducted to determine the relationship between colour characteristics, functional properties and thermal properties of heat moisture treated BGS (Tables 10, 11, 12). The correlation analysis revealed positive and negative relationships among the variables. Generally, a negative correlation is a relationship between two variables in which one variable increases as the other decreases, and vice versa. A positive correlation exists when one variable decreases as the other variable decreases or one variable increases while the other increases. In HMT 15, a correlation existed between WI and L*; ∆E and WI; ∆E and L*; H° and a*; *T*_*o*_ and *T*_*p*_; *T*_*p*_ and *T*_*c*_ and *T*_*o*_ and *T*_*c*_. However, there was no correlation between the thermal properties and colour characteristics except for *T*_*o,*_ which had a significant correlation with b*. Furthermore, it could be observed that WAC correlated with *T*_*o*_ and *T*_*p*_. A correlation between WI and L*; ∆E and WI; ∆E and L*; H° and a*, *T*_*o*_ and *T*_*p*_; *T*_*c*_ and *T*_*p*_; OAC and *T*_*c*_ was observed under HMT 25. However, there was no correlation between the thermal properties and colour characteristics (Table 11). Furthermore, thermal properties except for *T*_*c*_ correlated with solubility. Solubility is usually affected by higher temperatures and is increased by the collapsing of the starch granules by heating during HMT^60^. However, *T*_*o*_ is very low to have a notable effect on solubility. In HMT35, a correlation was observed between WI and L*; and WI; ∆E and L*; H° and a*; WAC and OAC; OAC and solubility; SP and *T*_*o*_; SP and *T*_*c*_; *T*_*o*_ and *T*_*p*_; *T*_*o*_ and *T*_*c*_; as well as between *T*_*p*_ and *T*_*c*_ (Table 12).

**Table 10:** Correlation matrix of colour, functional and thermal properties of HMT 15 Bambara groundnut starch.

	L*	a*	b*	C*	WI	∆E	H°	WAC (%)	OAC (%)	SP (g/g)	Solubility (%)	*T*_*o*_ (°C)	*T*_*p*_ (°C)	*T*_*c*_ (°C)	*∆H* (J/g)
L*	1														
a*	− 0.113	1													
b*	− 0.088	0.752*	1												
C*	− 0.094	0.860**	0.981**	1											
WI	0.989**	− 0.221	− 0.232	− 0.235	1										
∆E	− 0.963**	0.114	0.121	0.108	− 0.956**	1									
H°	0.076	− 0.734*	− 0.120	− 0.294	0.093	− 0.052	1								
WAC (%)	− 0.508	− 0.078	0.180	0.130	− 0.512	0.528	0.365	1							
OAC (%)	− 0.428	0.134	− 0.058	0.031	− 0.401	0.235	− 0.200	0.194	1						
SP (g/g)	0.281	0.306	0.008	0.100	0.264	− 0.322	− 0.459	− 0.514	− 0.121	1					
Solubility (%)	0.212	− 0.178	− 0.250	− 0.240	0.251	− 0.086	0.060	0.353	− 0.410	0.109	1				
*T*_*o*_ (°C)	0.349	− 0.362	− 0.642*	− 0.608	0.438	− 0.314	− 0.176	− 0.686*	− 0.103	0.262	0.084	1			
*T*_*p*_ (°C**)**	0.448	− 0.331	− 0.549	− 0.522	0.522	− 0.429	− 0.132	− 0.715*	− 0.059	0.257	0.007	0.979**	1		
*T*_*c*_ **(**°C**)**	0.517	− 0.244	− 0.248	− 0.243	0.549	− 0.558	0.072	− 0.56275	0.168	0.231	− 0.124	0.726*	0.842**	1	
∆H (J/g)	0.519	− 0.025	0.198	0.170	0.485	− 0.603	0.263	− 0.132	0.265	0.012	− 0.405	− 0.123	0.047	0.433	1

WAC: water absorption capacity; OAC: oil absorption capacity; SP: swelling power; ∆H: gelatinization enthalpy; ∆E: total colour difference; WI: whiteness index; L*: lightness/whiteness; a*: yellowness/redness; b*: blueness/greenness; C*: chroma; H°: hue; *T*_*o*_: onset temperature; *T*_*p*_: peak temperature; *T*_*c*_: conclusion temperature; ∆H: gelatinization enthalpy.

**Correlation is significant at the 0.01 level; *correlation is significant at 0.05 level.

**Table 11:** Correlation matrix of colour, functional and thermal properties of HMT 25 Bambara groundnut starch.

	L*	a*	b*	C*	WI	∆E	H°	WAC (%)	OAC (%)	SP (g/g)	Solubility (%)	*T*_*o*_ (°C)	*T*_*p*_ (°C)	*T*_*c*_ (°C)	∆H ( J/g)
L*	1														
a*	− 0.788**	1													
b*	− 0.854**	0.773**	1												
C*	− 0.876**	0.853**	0.983**	1											
WI	0.999**	− 0.811**	− 0.872**	− 0.895**	1										
∆E	− 0.924**	0.775**	0.846**	0.870*	− 0.925**	1									
H°	0.251	− 0.669*	− 0.077	− 0.213	0.268	− 0.164	1								
WAC (%)	0.057	− 0.188	− 0.105	− 0.140	0.073	0.080	0.145	1							
OAC (%)	0.312	− 0.027	− 0.164	− 0.191	0.308	− 0.155	− 0.163	0.425	1						
SP (g/g)	0.189	− 0.191	0.163	0.103	0.161	− 0.036	0.507	0.090	− 0.055	1					
Solubility (%)	0.321	− 0.377	− 0.210	− 0.257	0.324	− 0.301	0.387	− 0.346	0.269	0.209	1				
*T*_*o*_ (°C)	− 0.201	0.323	0.285	0.365	− 0.217	0.161	− 0.214	− 0.331	− 0.469	− 0.062	− 0.487	1			
*T*_*p*_ (°C)	− 0.287	0.369	0.368	0.436	− 0.305	0.252	− 0.180	− 0.249	− 0.581	0.034	− 0.637*	0.960**	1		
*T*_*c*_ (°C)	− 0.368	0.321	0.469	0.491	− 0.383	0.398	0.071	− 0.097	− 0.679*	0.273	− 0.659*	0.701*	0.863**	1	
∆H (J/g)	− 0.334	0.413	0.281	0.342	− 0.328	0.500	− 0.195	0.205	− 0.122	− 0.306	− 0.632*	0.421	0.497	0.570	1

WAC: water absorption capacity; OAC: oil absorption capacity; SP: swelling power; ∆H: gelatinization enthalpy; ∆E: total colour difference, WI: whiteness index; L*: lightness/whiteness; a*: yellowness/redness; b*: blueness/greenness; C*: chroma; H°: hue; *T*_*o*:_ onset temperature; *T*_*p*:_ peak temperature; *T*_*c*:_ conclusion temperature; ∆H: gelatinization enthalpy.

**Correlation is significant at the 0.01 level; *Correlation is significant at 0.05 level.

**Table 12:** Correlation matrix of colour, functional and thermal properties of HMT 35 Bambara groundnut starch.

	L*	a*	b*	C*	WI	∆E	H°	WAC (%)	OAC (%)	SP (g/g)	Solubility (%)	*T*_*o*_ (°C)	*T*_*p*_ (°C)	*T*_*c*_ (°C)	∆H (J/g)
L*	1														
a*	− 0.779**	1													
b*	− 0.722*	0.830**	1												
C*	− 0.773**	0.939**	0.969**	1											
WI	0.998**	− 0.807**	− 0.761*	− 0.809**	1										
∆E	− 0.950**	0.844**	0.662*	0.772**	− 0.951**	1									
H°	0.688*	− 0.946**	− 0.617	− 0.783**	0.706*	− 0.799**	1								
WAC (%)	− 0.754*	0.465	0.188	0.299	− 0.726*	0.755*	− 0.535	1							
OAC (%)	0.464	− 0.433	− 0.119	− 0.270	0.452	− 0.629	0.453	− 0.634*	1						
SP (g/g)	0.637*	− 0.770**	− 0.535	− 0.641*	0.647*	− 0.628	0.849**	− 0.511	0.123	1					
Solubility (%)	0.553	− 0.477	− 0.200	− 0.328	0.544	− 0.665*	0.512	− 0.592	0.757*	0.303	1				
T_o_ (°C)	0.321	− 0.664*	− 0.525	− 0.590	0.349	− 0.338	0.692*	− 0.211	0.094	0.786**	0.183	1			
T_P_ (°C)	0.089	− 0.389	− 0.134	− 0.226	0.107	− 0.196	0.491	− 0.277	0.335	0.525	0.454	0.748*	1		
T_c_ (°C)	0.271	− 0.539	− 0.351	− 0.424	0.289	− 0.281	0.604	− 0.243	0.134	0.683*	0.476	0.855**	0.793**	1	
∆H (J/g)	0.298	− 0.125	− 0.214	− 0.183	0.303	− 0.263	0.056	− 0.082	− 0.033	0.086	0.147	− 0.368	− 0.182	− 0.293	1

WAC: water absorption capacity; OAC: oil absorption capacity; SP: swelling power; ∆H: gelatinization enthalpy; ∆E: total colour difference, WI: whiteness index; L*: lightness/whiteness; a*: yellowness/redness; b*: blueness/greenness; C*: chroma; H°: hue; *T*_*o*_: onset temperature; *T*_*p*_: peak temperature; *T*_*c*_: conclusion temperature; ∆H: gelatinization enthalpy.

**Correlation is significant at the 0.01 level; *Correlation is significant at 0.05 level.

### Optimisation of heat moisture treated Bambara groundnut starch at various moisture levels

The independent variables and responses were kept in range as the criteria and constraints of optimisation. The results with the highest desirability were selected for each treatment. The optimum heat moisture treatment conditions for BG starch HMT 15, 25 and 35 were 80 °C for 30 min, 105.74 °C for 30 min and 113.16 °C for 30 min respectively. The predicted values of colour, functional and thermal properties for BGS HMT 15, 25 and 35 are reflected in Table 13. The desirability values of the obtained optimum conditions were 0.63 for HMT 15 and 1.00 for HMT 25 and 35, respectively.

**Table 13:** Predicted values of colour, functional and thermal properties of Bambara groundnut starch as affected by heat and moisture treatment.

Factors	HMT 15	HMT 25	HMT 35
Temperature	80	96.77	76.55
Time	30	104.16	81.65
Responses			
L*	82.08	81.18	79.79
a*	1.49	1.80	1.96
b*	3.16	3.65	4.62
C*	3.55	3.99	5.03
H°	65.16	64.23	66.55
WI	81.71	80.75	79.15
∆E	18.19	19.51	20.47
WAC (%)	189.27	185.65	162.90
OAC (%)	213.51	244.42	302.10
SP (g/g)	14.36	9.65	8.45
Solubility (%)	16.06	14.07	14.13
* T*_*o*_ (°C)	86.68	69.01	81.90
* T*_*p*_ (°C)	91.40	81.69	93.84
* T*_*c*_ (°C)	96.50	96.87	105.67
∆H (J/g)	5.36	11.20	5.66
Desirability	0.63	1.00	1.00

WAC: water absorption capacity; OAC: oil absorption capacity; SP: swelling power; ∆H: gelatinization enthalpy; ∆E: colour difference, WI: whiteness index; L*: lightness/whiteness; a*: yellowness/redness; b*: blueness/greenness; C*: chroma; H°: hue; *T*_*o*_: onset temperature; *T*_*p*_: peak temperature; *T*_*c*_: concluding temperature; ∆H: gelatinization enthalpy.

### Scanning electron microscopy of optimised heat-moisture treated Bambara groundnut starch

**Figure 3:**
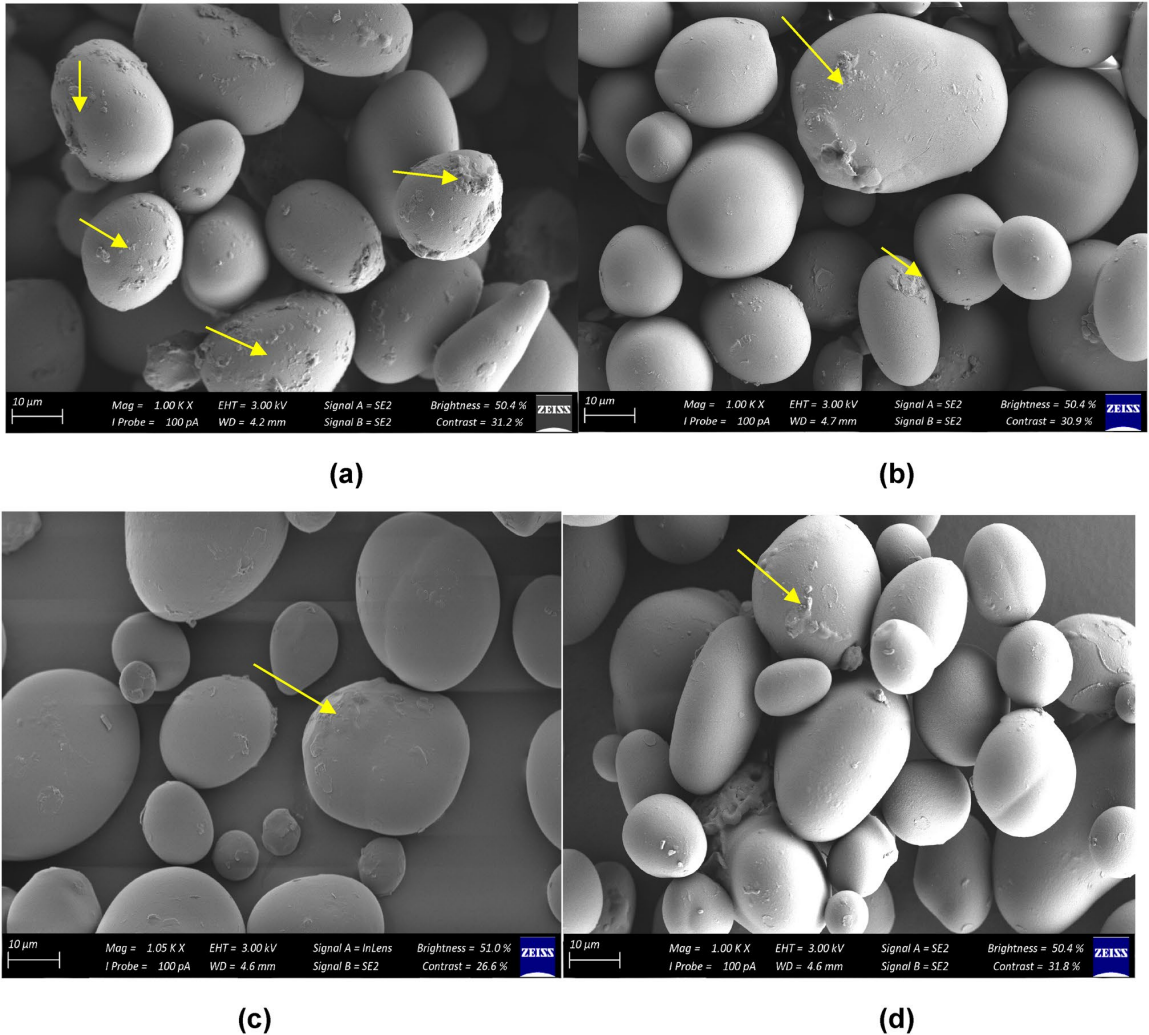
Micrographs of unmodified and heat moisture treated Bambara groundnut starch (BGS). (**a**) Scanning electron micrograph (×100) of unmodified BGS; (**b**) heat moisture treated at 15% moisture content Bambara groundnut starch (HMT 15-BGS); (**c**) HMT 25-BGS; and (**d**) HMT 25-BGS.

Scanning electron micrographs were obtained to observe the shape and surface characteristics of the unmodified and optimized HMT starches. Micrographs of the unmodified and optimised heat-moisture treated BGS granules at a magnification of 1.00× to 1.05× are shown in Fig. 3. The information obtained from the scanning electron micrograph indicates an oval and round shape for BGS granules with varying sizes. The range of the granule size width was between 4.2–4.7 mm and 10 μm for length. Adebowale et al*.*^61^ observed oval and round shapes for African yam bean starch, while Oyeyinka et al*.*^80^ observed spherical and round-shaped granules for microwave heated BGS.

The modified starches showed some changes in granule morphology as clumping of starch granules reduced with the application of HMT (Fig. 3b–d). Bambara groundnut starches showed cracked surfaces; however, the unmodified BGS presented a more agglomerated and broken surface than the heat moisture treated starches. The HMT starches exhibited more minor cracks and smoother surfaces as HMT conditions (moisture and temperature) increased. Kawabata et al.^81^ reported the development of cracks on the surface of treated maize and potato starches and the hollowing of starch granules. It has been suggested that cracks may be due to internal cracking occurring during air drying of the starch samples. Heat moisture treatment did not alter the size of the native starch. Generally, HMT has been reported to have no viable effect on the morphology of most starches such as cassava, potato, taro, cocoyam, finger millet and rice starches^60,63,82^. However, studies show that HMT decreases the relative crystallinity with an increase in the moisture of HMT starches^83^.

### Fourier transform infrared (FTIR) spectroscopy

**Figure 4:**
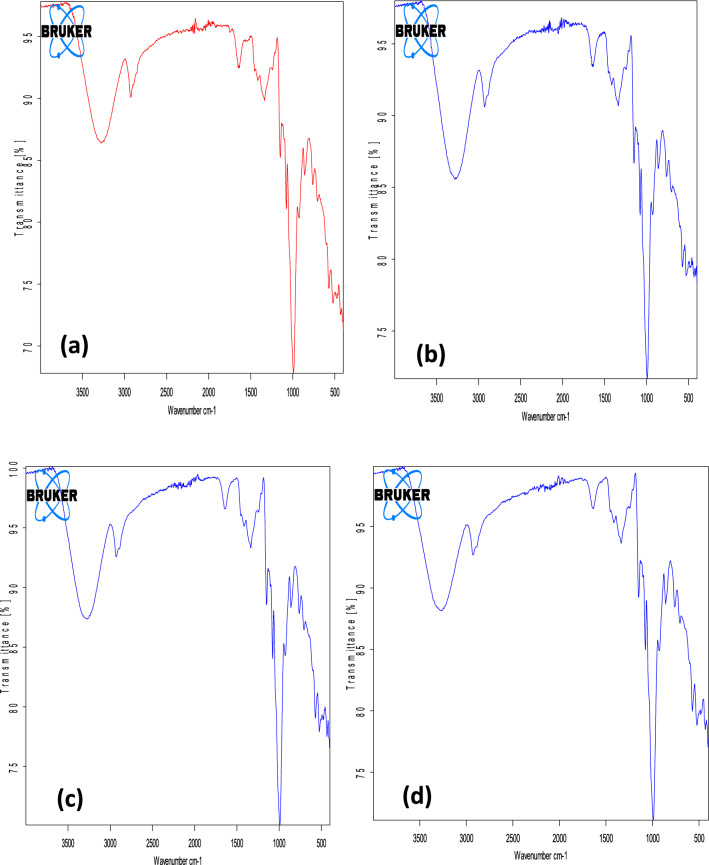
FTIR spectra of Bambara groundnut starch (BGS). (**a**) unmodified BGS; (**b**) Heat moisture treated at 15% moisture content Bambara groundnut starch (HMT 15-BGS); (**c**) HMT 25-BGS; and (**d**) HMT 35-BGS.

FTIR has been suggested to be sensitive to changes in structure on a molecular level (short-range order). Furthermore, it provides information on the structural arrangement of starch chains near the granule surface because the infrared beam penetrates only to a certain extent into the granule^64^. Unmodified and heat moisture treated BGS showed characteristic FTIR bands linked with common starches. All the samples displayed complex vibrations in the region below 1000 cm^−1^ due to the skeletal vibrations of the glucose pyranose ring. Broadband in the 3000–3600 region was observed with a peak at approximately 3425 cm^−1^ (Fig. 4). This peak could be attributed to OH stretching^41,84^. Similar FTIR band patterns were reported for different Bambara cultivar starches by Oyeyinka et al.^41^. In the C–H stretching region (2800–3000 cm^−1^), unmodified and HMT 15 showed lower peak intensities than HMT 25 and 35 BG starches. The variations in peak intensities could be related to the difference in amylose composition^85^. Other peaks were observed around 1650 cm^−1^ wavelength, which is probably associated with bending vibrations of H_2_O absorbed in the amorphous regions of the starch^41,64^. Similar observations were reported for potato, corn and wheat starches^85^.

## Conclusions

Heat-moisture treatment had a slight effect on the colour, functional and thermal properties of BGS. Reduced colour change and high L* values are desired in processed food, as these parameters can influence consumer acceptability. The enhanced oil absorption in (302.1) and reduced water absorption (162.9) of the HMT35-BG starch show desirable attributes in food production where starch is applied as a thickener. This is because of its ability to form firm gels, an advantage required in confectionaries and baked products as well as in the manufacture of noodles with greatly enhanced textural properties. Treatment time had a significant linear effect (p ≤ 0.05) on swelling power for HMT 15-BG starch. HMT 25 and HMT 35 both had the highest desirability value of 1.00 each. This result implies that processing BG starch at 96.77 °C & 104.16 min (HMT25) and 76.55 °C 81.65 min (HMT35) yielded optimal colour, functional and thermal properties. Information obtained from scanning electron micrograph indicates oval and round shapes for BGS granules, with varying sizes. The range of the granule size width was 4.2–4.7 mm and 10 μm for length. The unmodified starch presented a more agglomerated and cracked surface than the heat moisture-treated starches. The HMT starches seemed to have more minor cracks and a smoother surface was formed as HMT conditions (moisture and temperature) increased. Unmodified and heat moisture treated BGS showed characteristic FTIR bands linked with common starches. All the samples displayed complex vibrations in the region below 1000 cm^−1^ due to the skeletal vibrations of the glucose pyranose ring. The findings of this study will help food processors to tailor the process conditions of heat moisture treated BGS for industrial application and promote the diversified use of BG in the development of confectionaries, baked food products and noodles with enhanced texture properties.

## Supplementary Information

Supplementary Figures.

## Data Availability

Data will be made available on reasonable request. Data request can be made to VMM and TAA.
