# Supplementary material for: Optimisation of the techno-functional and thermal properties of heat moisture treated Bambara groundnut starch using response surface methodology
Source: Sci Rep. 2023 Feb 8;13:2261. doi: 10.1038/s41598-023-28451-0 (PMC9908914; doi:10.1038/s41598-023-28451-0)
Supplement: Supplementary file 1 — Supplementary Figures. [file 41598_2023_28451_MOESM1_ESM.docx]

**Supplementary Figures**

**(b)**

**(a)**

**(d)**

**(c)**

**Supplementary Fig. 1 (a-d)**: Contour response surface plots illustrating the effects of HMT treatment temperature and time on (a) = Onset temperature; (b) = Peak temperature; (c) = Concluding temperature; (d) = Enthalpy change of HMT 15-BG starch.

**(a)**

**(b)**

**(d)**

**(c)**

**Supplementary Fig. 2**: Contour response surface plots illustrating the effects of HMT treatment temperature and time on (a) = Onset temperature; (b) =Peak temperature; (c) = Concluding temperature (d) = Enthalpy change of HMT 25-BG starch.

**(b)**

**(a)**

**(d)**

**(c)**

**Supplementary Fig. 3**: Contour response surface plots illustrating the effects of HMT treatment temperature and time on (a) = Onset temperature; (b) = Peak temperature; (c) = Concluding temperature (d) = Enthalpy change of HMT 35-BG starch.
